# Perspectives of professionals participating in the Brazilian Network for the Surveillance of Severe Maternal Morbidity regarding the implementation of routine surveillance: a qualitative study

**DOI:** 10.1186/1742-4755-11-29

**Published:** 2014-04-08

**Authors:** Adriana Gomes Luz, Maria José Martins Duarte Osis, Meire Ribeiro, José Guilherme Cecatti, Eliana Amaral

**Affiliations:** 1 Department of Obstetrics and Gynecology, School of Medical Sciences, University of Campinas, Campinas, Brazil; 2 Campinas Center for Studies in Reproductive Health (CEMICAMP), Campinas, Brazil; 3 State University of Campinas - UNICAMP, Rua Alexander Fleming, 101, Cidade Universitária, 13083-881 Campinas, SP, Brazil

**Keywords:** Surveillance, Severe maternal morbidity, Professionals’ perspective, Vigilância, Morbidade materna grave, Perspectiva dos profissionais

## Abstract

**Background:**

The Brazilian Network for the Surveillance of Severe Maternal Morbidity was developed in Brazil with the participation of 27 centers in different regions of the country. The objective of the network project was to evaluate the frequency of severe maternal morbidity (near-miss and potentially life-threatening conditions) and the factors involved with these clinical conditions. Over the data collection period, this project implemented a surveillance system to identify these cases in the participating institutions. The objective of the present study was to evaluate the perspective of the professionals who participated in this network regarding the surveillance of cases of severe maternal morbidity, the facilities and difficulties encountered in involving colleagues in the process, and participants’ proposals to give continuity to this practice of qualifying maternal healthcare.

**Methods:**

A descriptive study with a qualitative approach was conducted in which coordinators, investigators and managers at all the 27 obstetric units participating in the network were interviewed. Data were collected at 6 and 12 months after implementation of the network during semi-structured telephone interviews that were recorded following verbal informed consent. Thematic content analysis was performed of the responses to the open questions in the interviews.

**Results:**

In the opinion of 60% of the participants, involving their colleagues in the surveillance process proved difficult, principally because these professionals were not very interested in the research project, but also because they found it difficult to review concepts and professional practices, because they had an excessive workload or due to operational and technical difficulties. The great majority considered that support from government agencies providing financial resources would be crucial to enable surveillance to be maintained or expanded and also to train a larger number of professionals and improve work conditions. The majority of participants found it difficult to define the ideal time interval at which surveillance should be conducted.

**Conclusion:**

The investigators, coordinators and managers involved in the Brazilian network project mentioned several problems that had to be confronted during this process; however, in their opinion the project should be maintained and even expanded in view of its potential to contribute towards improving obstetric care.

## Abstract (pt)

**Resumo:**

**Introdução**

A “Rede Nacional de Vigilância de Morbidade Materna Grave” (RNVMMG), foi desenvolvida no Brasil com a participação de 27 centros das diferentes regiões. O objetivo do projeto da RNVMMG era conhecer a frequência da morbidade materna grave (near miss e condições potencialmente ameaçadoras da vida) e os fatores envolvidos com estas condições clinicas. Esse projeto implantou, durante o período de coleta de dados, um sistema de vigilância destes casos nas instituições participantes. O objetivo do presente estudo era avaliar a perspectiva dos profissionais que participaram desta rede em relação ao processo de vigilância dos casos de morbidade materna grave, as facilidades e dificuldades para envolver os colegas e suas propostas para viabilizar a implantação definitiva desta pratica de qualificação dos serviços em saúde materna.

**Métodos**

Realizou-se um estudo descritivo, com abordagem qualitativa, no qual foram entrevistados coordenadores, pesquisadores e gestores de todas as 27 unidades obstétricas participantes da RNVMMG. Os dados foram coletados seis e 12 meses após o termino da implantação da RNVMMG através de entrevistas semiestruturadas por telefone, gravadas após consentimento informado verbal. Realizou-se analise temática de conteúdo das respostas as perguntas abertas constantes do roteiro de entrevista.

**Resultados**

Para 60% dos entrevistados foi difícil envolver seus colegas na vigilância devido, principalmente, ao fato de que os profissionais não se preocupavam muito com a pesquisa, dificuldade rever os conceitos e praticas profissionais, sobrecarga de trabalho do(a)s colegas e dificuldades operacionais e técnicas. A grande maioria considerou importante, para manter ou expandir a vigilância, o apoio de órgãos governamentais disponibilizando recursos financeiros também para qualificação dos profissionais e melhoria das condições de trabalho e, para a maioria dos participantes, e difícil definir um intervalo de tempo ideal para realização destes processos de vigilância.

**Conclusao**

Pesquisadores, coordenadores e gestores que estiveram envolvidos no projeto da RNVMMG no Brasil consideraram que se tratou de um processo em que se enfrentaram varias dificuldades, mas que deveria ser mantido e ate expandido, em vista de seu potencial de contribuição para melhorar a atenção obstétrica.

## Background

Healthcare systems worldwide are seeking better ways of responding to challenges to improve care. Maternal mortality and morbidity rates highlight the risk attributable to pregnancy and childbirth, as well as the performance of systems in terms of access to healthcare and the quality of the services provided. Nevertheless, performing a precise evaluation of these health indicators has proven problematic, since the indicators are not standardized [1,2]. The practical application of the concept of maternal near-miss has contributed significantly to improving the quality of obstetric care and has the potential to reduce maternal mortality and morbidity [3].

The term *near-miss* has been used historically to refer to a condition in which a woman suffers a severe complication, almost dies, but survives [1,4-8]. The World Health Organization (WHO) working group on maternal mortality and morbidity classifications considers the term *maternal near-miss* to be well-suited for describing this concept, and recommends its use by incorporating the analysis of such cases into the evaluation of the process of obstetric care, thus contributing considerably to ensuring that necessary measures are adopted to improve the quality of care [3].

In Latin America, maternal morbidity and mortality is a relevant problem despite the fact that WHO data show a reduction in the maternal mortality rate of approximately 60% between 1990 and 2008 [9]. In Brazil, maternal mortality rate showed 50% reduction between 1990–2008, achieving maternal mortality ratio of 67.8 in 2010 [10].

There is a lack of standardization in the criteria used to define severe maternal morbidity in population and hospital-based studies, with problems being found in the reporting of these conditions, both in the official records and by the women themselves [1,11-15].

As in other countries, the distribution of maternal mortality in Brazil is linked to disparities in socioeconomic development. The size of this country is also associated with cultural differences and socioeconomic inequalities, generating heterogeneity in the incidence of complications and in the ways of dealing with them [14].

Therefore, it was in this setting that the Brazilian Network for the Surveillance of Severe Maternal Morbidity was implemented, with the participation of 27 centers in different regions of the country. The initial objective of the project was to evaluate the frequency of severe maternal morbidity (near-miss and non near-miss [potentially life-threatening conditions]) and the factors associated with them [15], when introducing standard and unified criteria based on WHO guidelines for near-miss in a network of reference health facilities. This project also served to implement surveillance in the participating institutions, with all of the more severe cases occurring during the active period of the network being identified and analyzed until the patient was discharged from hospital.

The objective of the present study was to evaluate the perspective of the professionals who participated in this network regarding the process of surveillance in cases of severe maternal morbidity, the facilities and difficulties encountered in involving colleagues in the process, and the proposals of the participants of the Brazilian Network for the Surveillance of Severe Maternal Morbidity on how to give continuity to the surveillance process.

## Methods

A qualitative study was conducted in all 27 reference obstetric units of the Brazilian Network for the Surveillance of Severe Maternal Morbidity, located in various geographical regions of the country. The qualitative approach was in the form of a summative evaluation [16]. This approach was chosen because it allows identifying opinions and generalizations about how an intervention is effective, and under what conditions the efforts to the intervention implementation are successful [16].

The coordinator (responsible for identifying cases and collecting data), the principal investigator of the project (responsible for reviewing and confirming the data) and the service manager (the director of the obstetric unit or the clinical director) at each obstetric unit were invited to participate in the study. Of the 27 participating centers, all teaching hospitals, five were secondary level institutes and 22 were tertiary level, being 23 free-for-charge public institutions (four municipal, nine state, and 10 federal hospitals). With respect to the geographical location of the center, 12 were situated in the north, northeast or mid-west of the country, while 15 were in the south or southeast (data not presented as tables).

The interviews lasted about 10 minutes and were held at two moments: at six months (stage 1) and at 12 months (stage 2) after implementation of the network project. The interviews were conducted by telephone [17] and simultaneously recorded [18]. Interviewers skilled in how to conduct telephone surveys were specifically trained for this study. To guarantee anonymity, the recordings were identified only by numbers. Prior to initiating the interview, an informed consent form was read to the potential subjects and their consent, if given, was recorded.

Data collection was performed using an instrument containing a semi-structured section in which questions were asked regarding the facilities and difficulties encountered in involving colleagues in the project, and participants were asked for suggestions regarding how to ensure that the surveillance of cases of severe maternal morbidity would be maintained in their center. This instrument had been tested previously and some modifications were made following evaluation.

A total of 122 interviews were conducted at 6 and 12 months after the end of the activities involved in the Brazilian Network for the Surveillance of Severe Maternal Morbidity. Among the interviewees, 50% were women and 66% were over 40 years of age. The majority (86.7%) were physicians. At least six attempts were made to contact each potential subject by telephone before the interview was considered as lost. Even so, when telephone contact was unsuccessful, messages were sent by e-mail.

Of the 54 interviews scheduled to be conducted with investigators and coordinators in the first stage, 52 were indeed carried out. In the other two cases, one of the individuals in question was the principal investigator of the present study and the other was in hospital. Eight managers were also investigators/coordinators and 11 other managers could not be contacted or were unavailable due to a full agenda.

In stage 2, 48 of the 54 interviews planned with investigators and coordinators were completed. One interview with an investigator was discarded because the poor quality of the recording did not permit analysis to be made; another two investigators were unavailable for interviews during the interviewers’ working hours; and in another two cases contact proved impossible. Of the 27 managers scheduled to be interviewed, 10 were also investigators or coordinators and three possible subjects failed to reply to the request for an interview. The higher success rate in obtaining interviews in stage 2 was partly due to the fact that telephone contact had already been made previously with the clinical directors of each service.

The NVIVO® software program, version 9.0 was used to codify the interviews, organize and analyze the qualitative data. A thematic content analysis was performed. [16] The interviews carried out in stage 1 were transcribed in their totality and based on these interviews the topics discussed by the participants were identified and categories of analysis were defined. In principle, the categories were defined based on the study objectives and on the script used to conduct the interviews; however, categories that emerged during the interviews were also identified. In stage 2, the interviews were listened to in their entirety but only the segments referring to new categories were transcribed. This list of categories was used to compile a coding manual, which was then used to codify the transcripts and analyze the interviews conducted in both stages of the study. This process was performed by 2 co-authors (MJMDO and MR) and discussed and reviewed by the 2 others co-authors (AGL and EA). Quotations from the transcripts were used to illustrate the results presented.

This paper presents the findings of the following categories: the facilities and/or difficulties encountered in involving colleagues in surveillance, proposals on how to maintain or expand the project for periodic surveillance, and the ideal periodicity for surveillance.

## Results

Of the 54 interviews scheduled to be conducted with investigators and local coordinators of the Brazilian Network for the Surveillance of Severe Maternal Morbidity, 52 interviews were in fact carried out at 6 months and 48 at 12 months. With respect to the managers of the obstetric units, of the 27 individuals to be interviewed only eight were successfully contacted at 6 months and fourteen at 12 months.

### Facilities/difficulties encountered in involving colleagues in surveillance

The individuals interviewed were divided with respect to whether they had found it simple or difficult to involve their colleagues in the network project in each obstetric unit (Table 1).

**Table 1:** Facilities/difficulties encountered in involving colleagues in surveillance

**Factors that facilitated the involvement of colleagues in surveillance**	**Difficulties in involving colleagues in surveillance**
The project coordinator’s position of leadership within the center.	The residents and/or other professionals were not interested in the project.
The efforts of the team involved.	Reluctance of the doctors to agree to review concepts used routinely in their professional practice. A reluctance to change the ways in which they do things.
Clear and convincing explanations on the importance of the project.	Colleagues had an excessive workload
Center focused on research in maternal morbidity and mortality.	Operational and technical difficulties

The principal factors mentioned by the participants as having facilitated the involvement of their colleagues in surveillance are shown in Table 1 and in the following transcripts from their interviews. The most common answer given by the participants who considered that involving their colleagues had been easy was that their own position of leadership within the center had facilitated matters:

*“Yes, also because of my position as coordinator of the residency program; so by sensitizing the residents to the project, it was easier to get the professionals to conform with it”.* (Case 29, investigator).

The second most commonly mentioned reason was that the implementation of surveillance had been a team effort, involving the center as a whole.

*These objectives were shared by the entire medical and nursing staff and the purpose is to achieve greater cohesion within the center […] with respect to attitudes, insofar as this is possible.* (Case 25, investigator).

Some participants also mentioned that the fact that the center was in a teaching hospital and associated with research made it simpler to involve the professionals in the surveillance project:

*“Ah, I think that the very….since it is in a university center, I think that up to a certain point we were already doing this, because our group works a lot with maternal mortality, I think it complemented the care that we were already providing”.* (Case 49, investigator).

One participant emphasized that the quality of the network project was a decisive factor in facilitating the involvement of the team in this work:

*“I believe that because of the idea, the solidity of what it was, of how it was established and how the project was designed […] it really was a well-designed project and it never failed to motivate the people involved with it”.* (Case 36, investigator/manager).

The box in Table 1 lists the principal problems mentioned by participants as having hampered the involvement of colleagues in the surveillance project, with examples included from the interviews. The most commonly mentioned difficulty was that the residents and/or other professionals were not very interested in the project.

*“With the other staff members, it was very easy because they recognized the need to identify, to get to know the… a need to learn more about severe maternal morbidity emerged, lectures were given, classes, training everyone to use the process within the center, then the center was involved. Yes, I think that the residents were a little less involved because of their lack of knowledge, I think that immaturity was the problem and being unaware of the importance of the subject”.* (Case 21, coordinator).

One participant mentioned that a lack of human resources generates an excess workload in general and not just for the residents.

*“Look, there is a certain difficulty because of the problem that I spoke about earlier, which is the shortage of human resources. When you have a team working with fewer professionals than would be ideal, you have an excess workload, and when you have an excess workload, anything extra, any request, will always be met with a certain resistance from the professionals.”.* (Case 16, Manager).

Less commonly, participants reported operational and technical difficulties such as, for example, filling out medical charts or forms, even when the center is already used to doing so.

*“With any project, the difficulty is in filling out the paperwork. So, take the teaching hospital where we’re already used to that […] one more paper, yet another form, and we had no problem with that”.* (Case 28, investigator).

At another moment, a lack of commitment with research and a lack of knowledge on the subject were also mentioned.

*“It’s…I think it’s like this, more to do with personnel, principally older professionals, staff at the center who are not committed to the…to the teaching part, despite the fact that this is a teaching hospital. They believe that public service is just one more job and in my opinion, there is a lack of commitment and they just do things any old way”.* (Case 5, coordinator).

Although less common, the participants also reported that the professionals who began to be involved with surveillance found it difficult to revise the concepts of their professional practice, particularly with respect to allowing them to be evaluated and coordinated by others.

*“The difficulty that the doctors have in understanding that we have to review concepts all the time in our professional practice”.* (Case 24, Coordinator).

The difficulty in getting professionals to fill out medical charts in an appropriate way to obtain the data required for surveillance was also mentioned.

*“We had enormous difficulty getting them filled out, ensuring that colleagues filled out the patient charts adequately…the greatest difficulty is with filling out the charts”.* (Case 17, investigator).

A very few participants mentioned the difficulty caused by the lack of involvement of the board of directors of the institution with the project for political reasons.

Nevertheless, despite the difficulties mentioned above, the majority of the individuals interviewed considered that the project should be maintained or expanded to periodic surveillance. The proposals mentioned by the participants to ensure the continuity of the project are listed: constant monitoring and periodic surveys with audit of centers, linking local teams to a coordinating center, maintaining the procedures adopted during the project, training individuals to give continuity to the project, creation of a standardized form to enable the work to be managed better and political and/or institutional commitment.

Some of the interviewees reported that constant monitoring, periodic surveys or audits should be implemented at the centers to ensure the maintenance of the surveillance process or its expansion in a periodic pattern.

*“…I think that there are two principal strategies that could be considered: one refers to periodic surveys every two, three, five years, and the other is constant monitoring, right! I believe that evaluation in a survey conducted every few years would be more feasible because to do it daily as part of a routine is a bit difficult in my opinion”.* (Case 26, Coordinator).

The need for meetings to review protocols or reevaluate the impact of what is happening was also mentioned.

*“Ah, at least once a year, we need…we need to get together again and review all the protocols because evidence-based medicine changes very rapidly, so many things will change over time. [And how do you think this should be done?]. Well, I think that this group has already been formed, right? So there would be a periodic meeting of this same group, adding others, other elements, other professionals too”.* (Case 24, Coordinator).

Others spoke of maintaining and expanding the project by linking it to a research center that would manage it or to a government agency.

*“I think it’s like this, it would be much better to maintain contact…with institute X [the institute that had coordinated the network project]…with this group here from the project and not to lose this, this line of action, of feedback that we need to have. I think that in this way we would grow a lot and we are going to grow a lot”.* (Case 28, investigator).

Some participants mentioned the importance of the project experience as being essential for creating a permanent collaborative network of studies in perinatal health.

*“To form a network in obstetrics that…with several centers, in the most diverse regions,…the level of integration of these centers was…was…brilliant because…the people that were in this project perceived the importance of it for their centers and, principally, for the final care provided to their patients,…and we see that even after the data collection was complete, people are still enthusiastic, also regarding the introduction of new studies within the network.”.* (Case 2, Coordinator).

The need for training so that other professionals could be integrated into the teams, giving continuity to the surveillance, was also mentioned.

*“I think it is necessary to maintain this periodic surveillance. I am a member of the hospital’s Maternal Mortality Review Committee and one of our proposals within the committee is to maintain internal surveillance. We are training a postgraduate nursing student to give continuity to this service, exactly with the objective of maintaining surveillance and providing data within the center itself to generate conducts and projects to improve care”.* (Case 21, Coordinator).

Some of the participants also reported a need to create standardized forms to improve the way in which work is conducted in order to maintain surveillance. Some considered that there should be a nationwide electronic management system that could also be used as an evaluation system.

The need for political and institutional involvement from the managers of the institutions and also from the state and municipal health departments, as well as political will within the institutions were factors that were also deemed necessary to ensure the continuity and expansion of surveillance.

*“…I believe that the municipal health department has to be sensitized, the state health department has to be sensitized, to ensure that training is provided in these hospitals that are in actual fact still dealing with the situation of “where am I going to put the patient in labor since I have no beds”, understand? So from the time that you start to show that scales exist for evaluating morbidity, a protocol, then people begin to look at you, “but hold on, you haven’t yet helped me solve the most basic of the problems in here”, so I think that it’s….within the perspective that […] within the perspective in a university teaching hospital, I believe so.”.* (Case 60, Manager).

With respect to the periodicity of surveillance considered most appropriate, many different responses were received from the participants. In general, all considered that it is important to define this periodicity in order to clearly establish the follow-up of the surveillance process.

*“Periodicity, that is something that has to be considered, because it’s…if you reevaluate it in a very short time, then the effects of what you did are still very present, then you may imagine that…that everything has been resolved, while actually, if you wait a little bit longer that effect will begin to dissipate. So I don’t know exactly what the ideal periodicity would be…”.* (Case 2, Coordinator).

In general, the example in the preceding paragraph highlights the fact that the participants found it difficult to pinpoint a definite periodicity for surveillance.

Many of the participants suggested continuous surveillance on a daily basis; others suggested every three months. Some suggested increasing periodicity gradually to permit progressive compliance with routines:

*“I believe that evaluations should be made every three to six months, initially every three months, then this could be extended to six months, with the requirement always being that the medical team and the nursing team and the entire hospital team must report morbidity and mortality rates, I think this would make the medical team more aware”.* (Case 61, Manager).

Most of the participants considered a period of a semester or even one year as being ideal for showing the results within a unit. For some, a longer time interval would be more appropriate.

*“Look, I think that even for you to create....a culture of follow-up of this. I think that it could be an annual follow-up”.* (Case 49, Investigator).

## Discussion

In the opinion of the investigators, coordinators and managers who participated in implementing the Brazilian Network for the Surveillance of Severe Maternal Morbidity in Brazil, this was a process that involved various difficulties, but one that should be maintained and even expanded in view of its potential to contribute towards improving obstetric care.

Of the obstacles encountered during the process of implementing the network, the problems most commonly mentioned in this study included the doctors’ reluctance to agree to review concepts, colleagues’ excess workload, and operational and technical difficulties in complying with study protocols. The literature shows that implementing and maintaining an audit process represent complex procedures that require meticulous attention to an immense variety of components. It is important to evaluate and consider both the content and the context in which the audit occurs in order to develop strategies of sustainability [19,20].

We can consider that a limitation of this study was the focus on individual perspective. On the other hand, the individual perspective has the advantage to offer access to information that help explain the success or failure of the initiative, since it may inform about the process of care, and responsibilities of professionals involved. Since the individuals interviewed have a leadership role in their institutions, and these institutions represent the reference and teaching hospitals in their regions, it is important to value the potential for improvement highlighted, assuming all the difficulties to be confronted.

The need to base these changes in protocols or individual practices on evidence must be emphasized. Evidence-based medicine may be more readily incorporated when it is translated to attend the needs of the professionals who work within a specific context of the health system and when it is aligned with tacit knowledge that allows them to make decisions on a daily basis [21-23]. The declared objectives of evidence-based medicine include: (a) improving the quality of medical care, providing information on which to base clinical decisions; (b) guaranteeing that the care of the individual patient is based on the most current evidence available and on the best possible results; (c) encouraging physicians to maximize the likelihood of positive outcomes for many patients instead of just for the patient in question; and (d) minimizing the gap between research and practice [23].

Nevertheless, the transfer of knowledge to practice, i.e. the translation of the findings of research to health programs that will have an impact on health outcomes, is a complex and slow-moving process. Its determinants and the strategies for shortening the process and the time involved with it have been the subject of much debate and some studies. This field of research is known as translational research, with debates generally involving the transference of results from the bench to clinical practice [24].

Interventions to change the behavior of professionals and healthcare institutions in general, based on the best evidence from the biological sciences, but also from the human sciences, are the essence of this transdisciplinary field of knowledge. Without this understanding, obstacles would certainly be present, decelerating the progress towards improvements in maternal healthcare.

In this respect, one very positive aspect of the participants’ perspectives on this study is that the majority perceived the importance of maintaining or expanding the audit process in their center and throughout the country. They emphasized the relevance of the participation of coordinating centers, e.g. the Ministry of Health, in articulating the process, making resources available and probably in discussing the results. This perspective is coherent with reports in the literature that political support of the technical proposals is crucial to ensure that healthcare programs are successful [19,20].

Another relevant aspect for the participants in this study was the need to train the professionals while simultaneously paying attention to the problem of the excess workload in the obstetric care units. This is in agreement with the factors mentioned by the participants of this study as having facilitated implementation of the network project in their centers, factors that were associated with having a coordinator who was capable of leadership and a team that was aware of the importance of the project and synchronized with the culture of research to improve the care offered. Therefore, periodic audits are crucial, since they compel centers to review evidence that is constantly changing. This action may be associated with periodic reviews of the clinical management protocols, which are fundamental in providing guidance for professionals, in an attempt to improve the healthcare process. Indeed, the literature shows that in places in which the implementation of obstetric audits has led to an improvement in the quality of care, this success was attributed both to the care providers and to the decision-makers [25-28]. This reinforces the comments of many of the participants with respect to the need for political and/or institutional commitment to help implement the changes.

Interventions to change the behavior of healthcare professionals and institutions in general, based on the best evidence from biological sciences, but also from the human sciences, are the essence of this transdisciplinary field of knowledge. Without this knowledge, the process of qualifying maternal health would certainly be slower than expected.

## Abbreviations

WHO: World Health Organization.

## Competing interests

The authors declare that they have no competing interests.

## Authors’ contributions

The idea for the study arose in a group discussion among AGL, EA and JGC.The first version of the manuscript was drafted by AGL and EA, and then complemented with the suggestions of the others. AGL and MR were responsible for interviews. MR and MJMDO were responsible to define categories of analysis. All authors contributed to the development of the study protocol and approved the final version of the manuscript.
